# Prognostic value of 18F-FDG PET/MRI in patients with advanced oropharyngeal and hypopharyngeal squamous cell carcinoma

**DOI:** 10.1007/s12149-021-01590-y

**Published:** 2021-02-11

**Authors:** Leonardo Pace, Emanuele Nicolai, Carlo Cavaliere, Luca Basso, Nunzia Garbino, Giacomo Spinato, Marco Salvatore

**Affiliations:** 1 Dipartimento di Medicina Chirurgia e Odontoiatria “Scuola Medica Salernitana”, Università degli Studi di Salerno, via M. de Vito Piscicelli 44, 80128 Naples, Italy; 2 IRCCS SDN, Naples, Italy; 3 Dipartimento di Neuroscienze Sezione di Otorinolaringoiatria e Centro Regionale Tumori Testa Collo, Università degli Studi di Padova, Treviso, Italy; 4 Dipartimento di ChirurgiaOncologia e GastroenterologiaSezione di Oncologia ed Immunologia, Università degli Studi di Padova, Padua, Italy

**Keywords:** Oropharyngeal and hypopharyngeal cancer, PET/MRI, FDG, Overall survival

## Abstract

**Objective:**

The aim of this study was to evaluate the prognostic value of combined positron emission tomography (PET)/magnetic resonance imaging (MRI) parameters provided by simultaneous 18F-fluorodeoxyglucose (FDG) PET/MRI in patients with locally advanced oropharyngeal and hypopharyngeal squamous cell carcinomas (OHSCC).

**Methods:**

Forty-five patients with locally advanced OHSCC who underwent simultaneous FDG PET/MRI before (chemo)radiotherapy were retrospectively enrolled. Peak standardized uptake value (SULpeak), metabolic tumor volume (MTV) and total lesion glycolysis (TLG) of the primary lesion were obtained on PET data. On MRI scans, primary tumor size, diffusion and perfusion parameters were assessed using pre-contrast and high-resolution post-contrast images. Ratios between metabolic/metabolo-volumetric parameters and ADC were calculated. Comparisons between groups were performed by Student’s *t* test. Survival analysis was performed by univariate Cox proportional hazard regression analysis. Overall survival curves were obtained by the Kaplan–Meier method and compared with the log-rank test. Survivors were censored at the time of the last clinical control. *p* < 0.05 was considered statistically significant

**Results:**

During follow-up (mean 31.4 ± 21 months), there were 15 deaths. Univariate analysis shows that SULpeak and SULpeak/ADCmean were significant predictors of overall survival (OS). At multivariate analysis, only SULpeak remained a significant predictor of OS. Kaplan–Meier survival analyses showed that patients with higher SULpeak had poorer outcome compared to those with lower values (HR: 3.7, *p* = 0.007).

**Conclusion:**

Pre-therapy SULpeak of the primary site was predictive of overall survival in patients with oropharyngeal or hypopharyngeal cancer treated with (chemo)radiotherapy.

## Introduction

Head and neck squamous cell carcinoma (HNSCC), which is the most frequent malignancy of the upper aerodigestive tract, includes a variety of primary sites and is a heterogeneous entity. Among them, oropharyngeal and hypopharyngeal squamous cell carcinomas (OHSCC) are distinct in terms of etiology, lymphatic drainage, neural spread and treatment approach. Most patients with OHSCC have locoregionally aggressive tumors and advanced disease at presentation and are treated with (chemo)radiotherapy in the context of organ preservation. However, treatment outcome is frequently suboptimal and approximately 25% of patients still have residual disease after achieving a response to therapy. Thus, prognostic stratification is essential to optimize treatment and follow-up in non-metastatic OHSCC patients with stage III–IV disease.

Molecular imaging has a relevant clinical role in the evaluation of patients with head and neck cancer. Actually, both 18F-fluorodeoxyglucose (FDG) positron emission tomography/computed tomography (PET/CT) and Magnetic Resonance Imaging (MRI) have clinical value in the staging and prognostic evaluation of these patients [1–10]. In the last few years, integrated PET/MRI has been proposed in HNSCC [11–27] showing a diagnostic value at least similar to that of PET/CT and MRI. However, these studies have included patients with heterogeneous HNSCC and have focused mainly on diagnosis and staging. Therefore, our aim was to evaluate the prognostic value of FDG PET/MRI in patients with locally advanced OHSCC.

## Materials and methods

### Patient population

Patients were retrospectively selected from those with HNSCC undergoing FDG-PET/MRI. Inclusion criteria were: (a) locally advanced OHSCC; (b) FDG PET/MRI performed less than 2 months before start of therapy. A complete pretreatment work-up was performed in all patients and thereafter the patients were regularly followed at 1–3 months intervals. The study was approved by our Institutional Review Board authorization and Ethical Committee “IRCCS Pascale” and written informed consent was obtained from all subjects.

### Image acquisition and analysis

FDG-PET/MRI scans were acquired using a Biograph mMR (Siemens Healthcare. Erlangen. Germany) 81 ± 15 min after tracer injection. Briefly, PET data acquisition occurred for the first 7 min during MR acquisition and attenuation correction was obtained by means of 2-point Dixon 3-dimensional volumetric interpolated T1-weighted MRI sequences [17, 20, 23]. First, a coronal 2-point Dixon 3-dimensional volumetric interpolated breath-hold T1-weighted MRI sequence was acquired and used for the generation of attenuation maps and for anatomic allocation of the PET results. Then, MRI automatically generated 4 different images: T1-weighted in-phase, T1-weighted out-of-phase, water-only, and fat-only. Simultaneously with the start of the Dixon MRI sequence, PET acquisition started ensuring correct temporal and regional correspondence between MRI and PET data.

MRI protocol was performed with a 16-channel head and neck coil, including axial Fast Spin Echo (FSE) T2-weighted, axial FSE T1-weighted and axial DWI, obtained with a single-shot echo planar 2d SPAIR sequence using three b values: 0. 500 and 800 s/mm^2^. Perfusion studies were obtained during intravenous administration of a paramagnetic contrast agent (Magnevist. Bayer. Berlin. Germany) 0.2 ml/kg, with a flow rate of 3.5 ml/s, using a volumetric interpolated breath-hold examination (VIBE) dynamic sequence with 50 measurements (time resolution: 6 s). Two pre-contrast axial VIBE sequences with variable flip angles were obtained for T1 mapping. Finally, axial isometric high-resolution VIBE and axial Fast Field Echo (FFE) T1-weighted with fat-saturation sequences were acquired.

Peak standardized uptake value corrected for lean body mass (SULpeak), metabolic tumor volume (MTV) and total lesion glycolysis (TLG) of the primary lesion were obtained on PET data using a dedicated workstation and software (Syngo MMWP and SyngoTrueD; Syngo.via; SiemensMedical Solutions). SULpeak was derived automatically by the software using a spherical 1 cm^3^ volume-of-interest (VOI). Moreover, based on SULpeak values, a volumetric characterization of lesion burden was made, considering a metabolic tumor volume (MTV) with a threshold of 40% of the maximum signal intensity and TLG was obtained as MTV x SUVmean [20, 25].

On MRI scans, primary tumor size, diffusion and perfusion parameters were assessed using pre-contrast and high-resolution post-contrast images. The DCE-MRI images were post-processed on a workstation running commercially available software for tissue perfusion estimation (Tissue 4D, Siemens Medical Systems, Germany) [17, 20, 23]. Briefly, motion correction, pre-/post-contrast acquisitions co-registration, and T1 mapping were automatically performed, drawing a coarse ROI including the tumor and the neighboring vessels (carotid arteries, jugular vein). On the basis of Toft’s model, the following parametric maps were estimated: transfer constant between vascular and extravascular–extracellular space (EES) (Ktrans); the volume of EES (Ve); the transfer constant between EES and blood plasma (Kep); and the initial area under the concentration curve (iAUC). Free-hand ROI area values were positioned in major diameter lesion slice, avoiding necrotic areas and large feeding vessels, and then drawn with the same position and extent on each parametric map to extract Size maximum (as the largest diameter in the axial plane), Apparent Diffusion Coefficient (ADC), Ktrans, Kep, and iAUC mean values. Moreover, combined PET/MRI parameters (PET parameter corrected by tumor cellularity) were calculated as the ratio between metabolic/metabolo-volumetric PET parameters and ADC [21].

### Statistical analysis

MedCalc Statistical Software version 13.1.2 was used for statistical analysis (MedCalc Software. Ostend. Belgium; http://www.medcalc.org; 2014). Data are expressed as mean ± standard deviation. Comparisons between groups were performed by Student’s *t* test. Survival analysis was performed by univariate Cox proportional hazard regression analysis. Only variables showing a *p* value < 0.05 at univariate analysis were considered statistically significant and included in the multivariate analysis. Overall survival curves were obtained by the Kaplan–Meier method and compared with the log-rank test. Survivors were censored at the time of last clinical control. ROC analysis was used to find the threshold. A *p* < 0.05 was considered statistically significant.

## Results

**Fig. 1:**
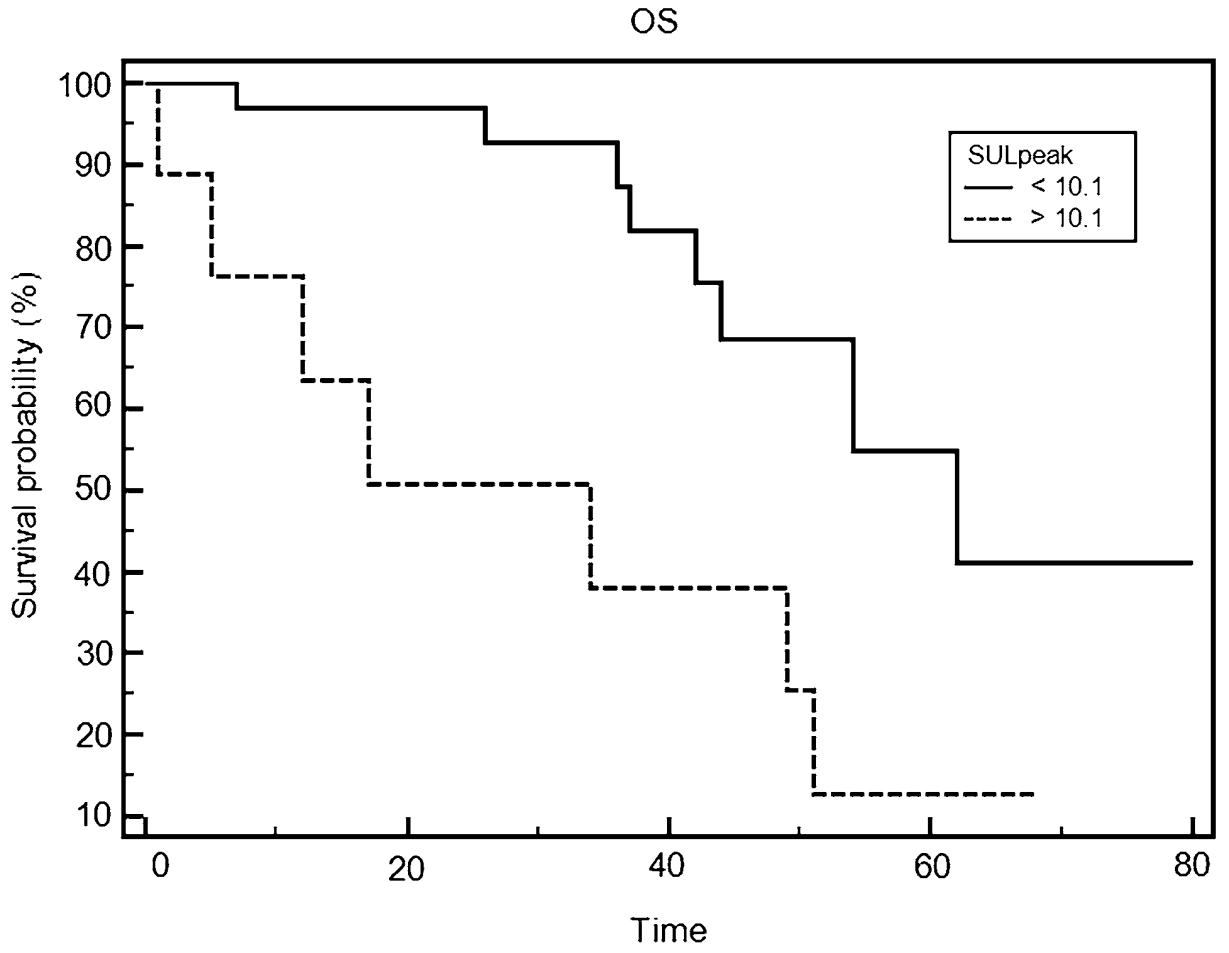
Overall survival (OS) by Kaplan–Meier analysis based on SULpeak. Hazard Ratio: 3.7. Log-rank test *p* < 0.01

A total of 45 patients with locally advanced OHSCC were included in the study (M/F = 38/7; mean age = 60 ± 11 years). TNM stages of the tumors were: stage III = 9; stage IV = 36. None of the imaging parameters, for either PET or MRI, differed significantly between patients in stage III or stage IV. During follow-up after (chemo)radiotherapy (mean 31.4 ± 21 months), there were 15 deaths. None of the clinical characteristics significantly differed between survivors and non-survivors, while among the imaging parameters SULpeak, iAUC, Ve, Sizemax, and SULpeak/ADCmean were significantly higher in non-survivors (Table 1). The univariate analysis (Table 2) showed that SULpeak and SULpeak/ADCmean were significant predictors of overall survival (OS). On the multivariate analysis, only SULpeak remained a significant predictor of OS. Kaplan–Meier survival analyses (Fig. 1) showed that patients with higher SULpeak had poorer outcome compared to those with lower values (HR: 3.7, *p* = 0.007).

**Table 1:** Comparison of clinical and imaging characteristics: survivors vs non-survivors

	Non-survivors (*n* = 15)	Survivors (*n* = 30)	*p* value
Gender (F/M)	2/13	5/25	0.884
Age (years)	64.7 ± 7.6	58.2 ± 12.1	0.07
Stage (III/IV)	2/13	7/23	0.6926
SULpeak	10.06 ± 4.67	6.99 ± 2.89	0.009
MTV	10.15 ± 9.47	9.25 ± 9.71	0.781
TLG	106.19 ± 98.81	96.48 ± 120.66	0.8
ADC mean	892.27 ± 292.96	892.15 ± 235.29	0.999
ADC min	771.11 ± 212.19	765.15 ± 239.83	0.939
Sizemax (mm)	38.27 ± 14.19	27.51 ± 14.21	0.021
Ktrans	311.15 ± 210.73	240.36 ± 106.36	0.1393
Kep	70.0 ± 23.93	72.68 ± 64.07	0.8768
Ve	390.95 ± 175.80	477.66 ± 116.93	0.05
iAUC	489.81 ± 621.07	54.17 ± 184.85	0.0009
Perfusion volume	14146.13 ± 12600.96	11682.48 ± 14005.35	0.5687
SULpeak/ADCmean	12.46 ± 6.02	8.92 ± 4.49	0.031
MTV/ADCmean	13.2 ± 14.64	12.81 ± 13.46	0.9295
TLG/ADCmean	137.2 ± 144.4	130.3 ± 158.2	0.888

**Table 2:** Univariate Cox proportional hazard regression analysis

Parameter	Chi square	*p* value
Gender	1.1	0.3
Age	1.6	0.2
Stage	0.9	0.3
SULpeak	19.8	0.0001
MTV	1.1	0.3
TLG	1.5	0.2
ADCmean	0.9	0.4
ADCmin	1.2	0.3
iAUC	3.6	0.06
Kep	0.9	0.4
Ktrans	0.884	0.3471
Perfusion volume	3.6	0.06
Sizemax	3.5	0.07
Ve	1.4	0.2
SULpeak/ADCmean	10.9	0.001
MTV/ADCmean	0.3	0.6
TLG/ADCmean	0.9	0.4

## Discussion

This study shows that in patients with locally advanced OHSCC both PET and MRI parameters obtained in the primary lesions are prognostic factors for overall survival.

Oropharyngeal and hypopharyngeal cancer is regarded as a comparatively rare disease. Primary oropharyngeal and hypopharyngeal squamous cell carcinomas (OHSCC) are distinct among head and neck cancers in terms of etiology, lymphatic drainage from adjacent organs, and treatment approach The National Comprehensive Cancer Network (NCCN) guidelines note some differences for each disease, but treatment selection is broadly similar for these cancers, and thus we evaluated patients with oropharyngeal and hypopharyngeal squamous cell carcinomas together in this study.

Although several papers dealing with PET/MRI in HNSCC have been published in the last few years [11–27], most of them have focused on diagnosis and staging in patients with various HNSCC and only a few have dealt with prognosis or therapy response assessment [7, 8, 21, 23]. Martens et al. [7] demonstrated that pretreatment DWI and FDG-PET parameters have predictive value for treatment failure and death. Similarly, Kim et al. [21] found that PET/MRI parameters could be effective predictors of tumor treatment failure. Both studies included patients with different types of HNSCC, and it is well known that HNSCC is a heterogeneous group of cancers with different characteristics in terms of presentation and prognosis [26]. Moreover, it has been demonstrated that the predictive efficacy of pretreatment 18F-FDG PET/CT varies with different primary sites [4]. In the present study, we included only patients with locally advanced OHSCC, most of them being in stage IV. In this selected group of patients, we found that both MRI (Size max, Ve, and iAUC) and PET (SULpeak) parameters were significantly different between survivors and non survivors, as well as a combined parameter, i.e. SULpeak/ADCmean. However, only SULpeak and SULpeak/ADCmean were significant predictors of overall survival, with SULpeak being the only independent prognostic indicator on multivariate analysis. This result is in line with previous studies using PET/CT showing that uptake of the primary site is a significant prognostic predictor in HNSCC as well as in OHSCC [27, 28]. In our study, neither MTV nor TLG were different between survivors and non survivors and they were not prognostic indicators, as previously reported [7, 8, 21]. The different results observed could be due to differences in the population studied, since in the present study locally advanced OHSCC constituted the study group, while in the other studies, patients with various HNSCC in different stages were studied.

Although in our study, some MRI-derived parameters (namely iAUC, Ve, and Size max) were significantly different between survivors and non survivors, none of them was a significant predictor of overall survival. Previous studies have reported conflicting results in terms of the prognostic role of MRI [7, 8, 21]. In particular, Kim et al. did not find any prognostic role for MRI in patients with HNSCC undergoing surgery, while the ratio between metabolic/metabolo-volumetric PET parameters and ADC were predictors of disease-free survival [21]. On the other hand, predictive value for treatment failure of quantitative diffusion-weighted imaging and PET in HNSCC treated by (chemo)radiotherapy was observed in one study [7], and DCE-MRI parameters showed a prognostic value for survival in OHSCC treated with (chemo)radiotherapy [8]. Moreover, Ng et al. identified MR-perfusion (Kep-tumour, Ve-node) and PET-metabolic (SUVmax-tumour) parameters as independent prognosticators for OHSCC treated with chemoradiation, suggesting their combination into a prognostic scoring system for survival stratification [29]. In our study, ADC has no prognostic role, and this seems in contrast with other studies using DWI to predict response to chemoradiotherapy in patients with HNSCC, albeit with conflicting results [7, 8, 21] and with its interpretation as an indicator of the cellularity of the malignancy. Moreover, it should be noted that similar results have been found by others [8, 29] in evaluating the prognostic power of ADC in oropharyngeal or hypopharyngeal squamous cell carcinoma. Thus, it appears that no definite conclusion can be drawn on the prognostic role of MRI component of PET/MRI. Furthermore, advanced MR sequences such as intravoxel incoherent motion or chemical exchange saturation transfer imaging are currently developing. Although recent evidence has demonstrated their role in HNSCC characterization and staging, a possible application in predicting survival remain unexplored (30, 31).

Some limitations of this study should be acknowledged: its single-institution retrospective design, a relatively small sample size to derive definite conclusions (although a sufficient median duration of follow-up was employed), and the absence of data human papillomavirus status.

In conclusion, in this study, pre-therapy SULpeak of the primary site was predictive of overall survival in patients with oropharyngeal or hypopharyngeal cancer treated with (chemo)radiotherapy.
